# Comparative performance and external validation of the multivariable PREDICT *Prostate* tool for non-metastatic prostate cancer: a study in 69,206 men from Prostate Cancer data Base Sweden (PCBaSe)

**DOI:** 10.1186/s12916-020-01606-w

**Published:** 2020-06-16

**Authors:** David Thurtle, Ola Bratt, Pär Stattin, Paul Pharoah, Vincent Gnanapragasam

**Affiliations:** 1 Academic Urology Group, University of Cambridge, Norman Bleehan Offices, Addenbrookes Hospital, Hills Road, Cambridge, CB2 0QQ UK; 2 Department of Urology, Institute of Clinical Science, Sahlgrenska Academy, University of Gothenburg, and Sahlgrenska University Hospital, Region Västra Götaland, Gothenburg, Sweden; 3 Department of Surgical Sciences, Uppsala University, Uppsala, Sweden; 4 Department of Cancer Epidemiology, University of Cambridge, Cambridge, UK; 5 Vincent Gnanapragasam, Academic Urology Group, University of Cambridge, Cambridge, UK

**Keywords:** Prostate cancer, Prognosis, Prostate cancer-specific mortality, PCSM, Survival, Overall mortality, Competing risks, Decision aid

## Abstract

**Background:**

PREDICT *Prostate* is an endorsed prognostic model that provides individualised long-term prostate cancer-specific and overall survival estimates. The model, derived from UK data, estimates potential treatment benefit on overall survival. In this study, we externally validated the model in a large independent dataset and compared performance to existing models and within treatment groups.

**Methods:**

Men with non-metastatic prostate cancer and prostate-specific antigen (PSA) < 100 ng/ml diagnosed between 2000 and 2010 in the nationwide population-based Prostate Cancer data Base Sweden (PCBaSe) were included. Data on age, PSA, clinical stage, grade group, biopsy involvement, primary treatment and comorbidity were retrieved. Sixty-nine thousand two hundred six men were included with 13.9 years of median follow-up. Fifteen-year survival estimates were calculated using PREDICT *Prostate* for prostate cancer-specific mortality (PCSM) and all-cause mortality (ACM). Discrimination was assessed using Harrell’s concordance (c)-index in R. Calibration was evaluated using cumulative available follow-up in Stata (TX, USA).

**Results:**

Overall discrimination of PREDICT *Prostate* was good with c-indices of 0.85 (95% CI 0.85–0.86) for PCSM and 0.79 (95% CI 0.79–0.80) for ACM. Overall calibration of the model was excellent with 25,925 deaths predicted and 25,849 deaths observed. Within the conservative management and radical treatment groups, c-indices for 15-year PCSM were 0.81 and 0.78, respectively. Calibration also remained good within treatment groups. The discrimination of PREDICT Prostate significantly outperformed the EAU, NCCN and CAPRA scores for both PCSM and ACM within this cohort overall.

A key limitation is the use of retrospective cohort data.

**Conclusions:**

This large external validation demonstrates that PREDICT *Prostate* is a robust and generalisable model to aid clinical decision-making.

## Background

Prostate cancer represents a growing burden on health care globally, with increasing numbers and proportions of men presenting with non-metastatic prostate cancer (PCa) [1]. Alongside this, there has been increased confidence in the use of conservative management (active surveillance and watchful waiting) [2]. Understanding disease prognosis to guide treatment decision-making is therefore of great importance. However, until recently, no high-quality individualised model for survival existed.

Using data from over 10,000 UK men, we have previously published an individualised prognostic model for cancer-specific and overall survival called ‘PREDICT *Prostate*’ [3]. PREDICT *Prostate* (available online [4]) provides cancer-specific and overall percentage survival estimates for up to 15 years and has been endorsed by the National Institute for Health and Care Excellence (NICE) [5]. To maximise usability, it uses routinely available clinico-pathological data (age, PSA, grade, stage, biopsy involvement, treatment type and comorbidity). It represents real-world data from a non-screened, primary diagnostic cohort, including a significant number of men treated conservatively. Crucially, the model also allows adjustment for competing mortalities by incorporating both cancer-specific and non-cancer survival outcomes to contextualise the diagnosis as part of a decision aid. Internal validation and accuracy within a small external population were promising during model development [3]. However, external validation in independent cohorts, ideally in a different location, is vital to demonstrate generalisability and accuracy of a multivariable prognostic model [6].

The Prostate Cancer data Base Sweden (PCBaSe) is one of the largest and most comprehensive prostate cancer cohorts world-wide and is well suited for external validation of PREDICT *Prostate* [7]. The aim of this study was to validate PREDICT *Prostate* and compare performance to existing models.

## Methods

### Source of data

Data from PCBaSE 3.0 were used, according to a pre-specified project outline (Additional file 2) [8–10]. PCBaSe was created by the combination of the National Prostate Cancer Register of Sweden with other national healthcare and demographic databases [11]. The capture rate of this register is 98% of all incident prostate cancer cases compared to the Swedish Cancer Registry—to which registration is mandated by law [12]. The cause of death information is updated from the Cause of Death Registry which captures all deaths in Sweden. The agreement between the recorded cause of death and reviewed medical records has been reported at 86% (95% CI 85–87%) [13].

### Participants and predictors

We included men within PCBaSe diagnosed with PCa between 1 January 2000 and 31 December 2010, with no evidence of metastatic disease and prostate-specific antigen (PSA) < 100 ng/ml. Cases were censored at death, migration or 31 December 2016, whichever event occurred first. Data were available for 82,936 men. Outcome events were ‘PCa death’ or ‘any-cause death’ from which ‘non-PCa death’ was derived. Intact data were required for variables mandatory within the model: age, PSA, T stage, histological grade group, primary treatment type and comorbidity. This led to the exclusion of 13,730 (16.6%) cases, leaving a final analysable dataset of 69,206 (Table 1). Missing data were most abundant for the histological grade group (*n* = 8117), as primary and secondary Gleason grades were not always registered. Data were also missing on PSA (*n* = 2124), T stage (*n* = 1364), age (*n* = 4) and primary treatment (*n* = 3960). Some men had missing data for more than one variable. All variables were determined at the time of diagnosis. Biopsy characteristics are an optional variable in the PREDICT *Prostate* model; therefore, missing data on proportion of positive cores ([PPC] = number of cores with any cancer/number of cores taken) were tolerated. We also re-tested the value of PPC to predict PCa death in a sub-group with intact biopsy information (*n* = 44,163) using the same method as previously [3]. Primary treatment was defined as the radical treatment received up to 12 months after the date of diagnosis, or conservative management. The same definition of comorbidity was used as in the model development: the combination of both Charlson Comorbidity Index of 1 or greater (excluding PCa) and a hospital admission in the 2 years preceding PCa diagnosis [3]. Up to 2008, the treatment strategies of active surveillance and watchful waiting were reported as conservative management. After 2008, these strategies were registered as separate entities. We used conservative management as a treatment strategy also for men diagnosed after 2008, although a small, well-defined active surveillance group was separately analysed.

**Table 1:** Baseline cohort characteristics in the original UK model development cohort and Prostate Cancer data Base Sweden (PCBaSe) cohort

	UK model development cohort		Sweden PCBase cohort	
**Total subjects**	7063		69,206	
**Time at risk (years)**	58,138		589,733	
		Range		Range
**Median follow-up (years)**	9.8	0–16	13.9	0–17
**Age** (mean, SD)	69.9	8.34	68.8	8.83
**PSA** (mean, SD)	18.5	17.5	15.7	17.0
**Grade groups**		%		%
1	2317	32.8	36,992	53.5
2	2125	30.1	14,015	20.3
3	1057	15.0	7774	11.2
4	710	10.1	6345	9.2
5	854	12.1	4080	5.9
**T stage**				
1	3761	53.2	35,700	51.6
2	2270	32.1	22,478	32.5
3	977	13.8	10,295	14.9
4	55	0.8	733	1.1
**Primary treatment**				
Radical prostatectomy	995	14.1	20,936	30.3
Radical radiotherapy	2457	34.8	11,906	17.2
Androgen deprivation monotherapy	2226	31.5	15,980	23.1
Conservative management	1385	19.6	20,384	29.5
**Comorbidity**				
No recorded comorbidity	6363	90.1	62,173	89.8
Comorbidity (Charlson Score ≥ 1)	700	9.9	7033	10.2
**10-year outcomes**				
PCa death	712		6993	
Non-PCa death	1555		15,122	
Any-cause death	2267		22,115	
**Overall outcomes**				
PCa death	846		8151	
Non-PCa death	1829		18,003	
Any-cause death	2675		26,154	
**Crude PCS mortality rate (per patient year)**	1.46		1.38	
**Annual overall mortality rate (per patient year)**	4.60		4.43	

*PCa* prostate cancer, *SD* standard deviation

### Outcome

The model estimates prostate cancer-specific mortality (PCSM), non-PCa mortality (NPCM) and overall or all-cause mortality (ACM), counted from the time of diagnosis. It provides estimates following conservative management and radical treatment (by either radical prostatectomy or radiotherapy).

### Statistical analysis methods

Beta coefficients for each prognostic factor in the model were applied to derive prognostic indices for PCSM and NPCM for each patient. These were used in combination with the model’s baseline hazard functions and time at risk to create individual estimates of unadjusted PCSM and NPCM over 15 years. These estimates were adjusted for the competing risks between the two causes of death to generate ACM estimates. To assess discrimination, 15-year estimates were generated. Harrell’s concordance index (c-index) was then applied using the ‘Hmisc’ package in R [14]. Discrimination using PREDICT *Prostate* was compared to the EAU and NCCN stratification systems and the UCSF CAPRA score [15–17]. Sub-classification of stage T2 was not available; therefore, T2 was assumed to be T2a for the sake of these classifications. When PPC was unknown, it was assumed to be < 34% in the CAPRA model. Adjusted predictions of cumulative PCSM, NPCM and ACM were generated using the available follow-up for the assessment of model calibration. Calibration was assessed using a chi-square goodness of fit (GOF) across quintiles of risk using the method of May and Hosmer [18]. Calibration was also assessed within treatment sub-groups. All data analyses were performed in Stata™ 14, unless otherwise stated above.

## Results

### Participants

Sixty-nine thousand two hundred six men were included with 13.9 years of median follow-up. The Swedish population attributes at baseline are compared to the UK model development cohort in Table 1. Patient characteristics were similar in both cohorts, with a larger proportion of grade group 1 disease in the Swedish cohort. A larger proportion of men underwent surgery as opposed to radiotherapy in the Swedish cohort, and smaller proportion were treated with primary androgen deprivation therapy in this time period. Breakdown of the patients by risk groups is reported in Additional file 1: Table S1.

### Model performance

**Fig. 1:**
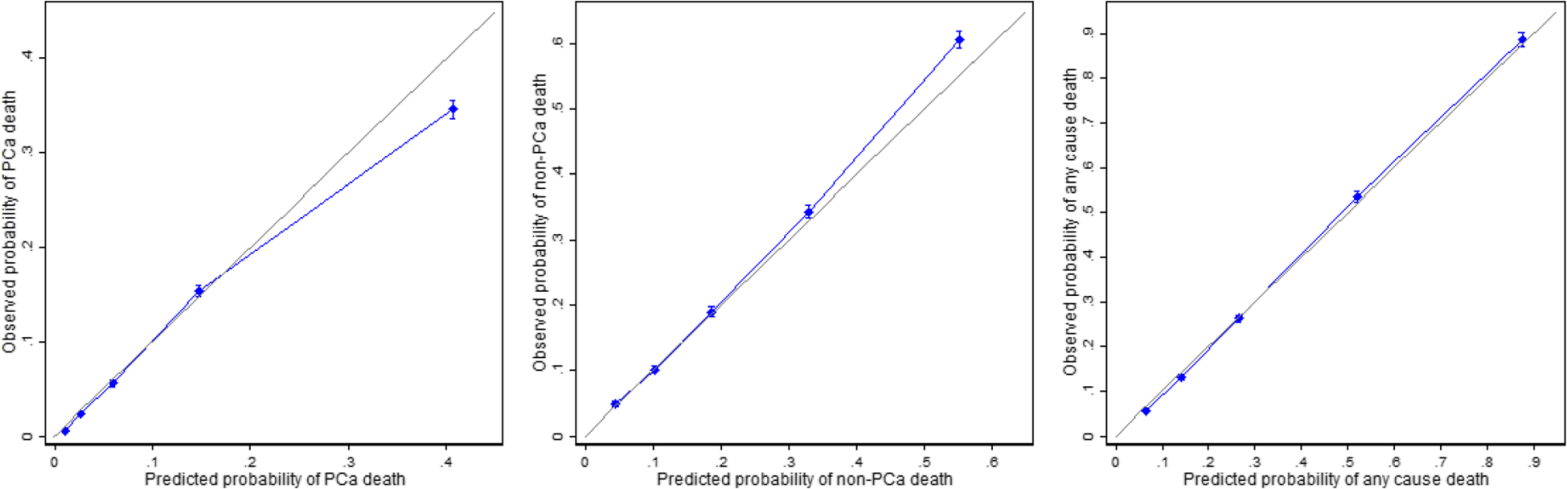
Calibration curves demonstrating observed and expected 15-year probability of death across quintiles or risk for prostate cancer (PCa) death (left), non-PCa death (centre) and any-cause death (right)

Overall discrimination of PREDICT *Prostate* was very good with c-indices 0.85 (95% CI 0.85–0.86) for PCSM and 0.79 (95% CI 0.79–0.79) for ACM (Table 2). Overall calibration of the model was excellent with 25,925 deaths predicted and 25,849 deaths observed in PCBaSe. This equates to an overall observed to expected (O:E) ratio of 1:1.003. Calibration across quintiles of risk is shown in Fig. 1 and Additional file 1: Table S2. Although the O:E ratio for any-cause death was very close to 1, expected numbers of PCa deaths were slightly higher than observed (O:E 0.897) and expected numbers of non-PCa deaths were lower than observed (O:E 1.060), particularly in the highest risk quintiles.

**Table 2:** Discrimination of PREDICT Prostate (PREDICT) within treatment sub-groups and comparison to other existing tools

			PCSM			Overall		
	*N*	Tool	c-index	SD	*p*	c-index	SD	*p*
Conservative management	**20,384**	**PREDICT**	**0.810**	**0.010**		**0.740**	**0.0057**	
	**20,384**	EAU	0.746	0.0115	**< 0.001**	0.636	0.0061	< 0.001
	**20,384**	NCCN	0.760	0.0118	**< 0.001**	0.643	0.0063	< 0.001
	**20,384**	CAPRA	0.765	0.0125	**< 0.001**	0.643	0.0064	< 0.001
Radical treatment	**32,842**	**PREDICT**	**0.784**	**0.0122**		**0.670**	**0.0077**	
	**32,842**	EAU	0.742	0.0113	**< 0.001**	0.606	0.0077	**< 0.001**
	**32,842**	NCCN	0.769	0.0106	**0.063**	0.617	0.0081	**< 0.001**
	**32,842**	CAPRA	0.780	0.0116	**0.475**	0.625	0.0082	**< 0.001**
**Overall**	**69,206**	**PREDICT**	**0.852**	**0.0038**		**0.792**	**0.0028**	

*EAU* European Association of Urology criteria, *NCCN* National Cancer Care Network criteria, *CAPRA* UCSF Cancer of the prostate risk assessment criteria, *SD* standard deviation

### Treatment sub-groups

Overall, 20,384 men underwent conservative management and 32,842 received radical treatment. Within these groups, c-indices remained good, with c-index for 15-year PCSM 0.81 (95% CI 0.80–0.82) for conservative management and 0.78 (95% CI 0.77–0.80) for radical treatment (Table 2).

Among men on well-defined active surveillance, c-indices were further improved at 0.88 for PCSM and 0.75 for ACM (Additional file 1: Table S3). Calibration also remained good within treatment groups with differences between observed and predicted numbers of overall deaths 1.4%, 2.2% and 3.1% among men who received active surveillance, radiotherapy and prostatectomy, respectively (Table 3). The model overestimated PCSM and underestimated NPCM within the sub-group which received androgen deprivation monotherapy by as much as 8%—but remained within 2% for overall death (Table 3).

**Table 3:** Calibration of PREDICT *Prostate* mortality estimates with observed numbers of deaths within treatment groups

		PCa death			Non-PCa death			Any-cause death		
	***n***	Obs	Pred	% Diff	Obs	Pred	% Diff	Obs	Pred	% Diff
**‘Active surveillance’**	6224	195	191	0.06	850	940	1.44	1045	1131	1.38
**‘Watchful waiting’**	2745	239	198	1.49	942	915	0.98	1181	1112	2.51
**‘Other conservative’**	11,415	1358	1373	0.13	4906	4535	3.25	6264	5908	3.12
**Radical prostatectomy**	20,936	550	703	0.73	1919	2403	2.31	2469	3107	3.05
**Radiotherapy**	8953	737	560	1.94	1591	1594	0.03	2318	2155	2.18
**ADT**	15,980	4809	5798	6.19	7215	5993	7.65	12,024	11,792	1.45

### Comparison to existing models

PREDICT *Prostate* significantly outperformed the comparator models when predicting ACM, both overall and within every major treatment sub-group (Table 2 and Additional file 1: Table S3). Discriminatory performance was significantly better for PCSM overall (Additional file 1: Table S4). Across all treatment sub-groups, the model outperformed the 3-stratum EAU risk categories. Improvements in discrimination failed to reach significance for PCSM in some comparisons with the NCCN and CAPRA scores, but in only one incidence was the c-index better for one of these comparator models (CAPRA score for PCSM among RP patients, Additional file 1: Table S3).

### Biopsy parameter sub-analysis

Biopsy parameterisation using percentage of positive cores (PPC) was re-explored within a group of 44,163 men who had this information registered (Additional file 1). Inclusion of biopsy characteristics did not significantly alter the discriminatory performance of the model (Additional file 1: Tables S4 & Table S5): either using a dichotomous 50% percentage of cores cut-off or PPC as a continuous variable. Inclusion of biopsy information did improve calibration across lower-risk quintiles of risk for PCSM. Calibration for any-cause death however was unchanged regardless of inclusion of biopsy information (Additional file 1: Table S6 & Figure S1).

## Discussion

In this large external validation cohort, we demonstrated that PREDICT *Prostate* is a robust and generalisable long-term prognostic model. In the analysis of an independent cohort, ten times larger than the original cohort, discriminatory accuracy and calibration was good. This also remained true within treatment groups, particularly in men managed conservatively or by radical therapy.

Conveying information to an individual about their disease prognosis within their own context of competing mortality has historically been an imprecise exercise with little objective data available. The most current prognostication is based on stratification groups of the cancer itself and discussions with clinicians who may be conflicted towards a particular treatment [19–21]. PREDICT *Prostate* was conceived to address this gap in clinical need and standardise the decision-making process [3] and has shown promise to positively influence clinical decision-making [22]. It is built around long-term actual survival data and has been designed to address all AJCC criteria [6].

In the model development study, c-indices were 0.84 for PCSM and 0.77 for ACM within the UK validation cohort [3]. In the original study, external validity was also assessed within a Singaporean cohort. However, this cohort was small (*n* = 2546) and follow-up was quite short (5.1 years). Here we show in a cohort of > 69,000 men with longer median follow-up that our c-indices were actually improved to 0.85 for PCSM and 0.79 for ACM with excellent calibration. We did note a marginal overestimation of PCSM, which was contrary to the slight underestimation we had observed in the Singapore external validation in the original paper [3]. Given that the model is very well calibrated for ACM, this apparent overestimation of PCSM (and corresponding underestimation of NPCM) is likely to be a result of differences in cause of death classification, reporting or recording practices. ACM is the key outcome of interest, and a more unequivocal endpoint, against which this model performs very well.

When compared to existing models, PREDICT *Prostate* consistently outperformed the three-stratum risk classification system used in the EAU, D’Amico and NICE stratification criteria [16, 20, 23]. We recognise that comparisons against these risk stratification criteria are limited and that they are not designed to be prognostic nomograms; however, they are widely used in clinical practice to inform treatment decisions. Benefits of PREDICT were also seen against the NCCN and CAPRA scores, which add more granularity but ultimately retain a grouping system rather than individual estimates [16, 17]. For the outcome of PCSM, the CAPRA score did perform similarly well for some treatment groups, particularly in men treated with prostatectomy. This is unsurprising, as the model was originally built around prostatectomy patients [24]. It should be noted that PREDICT *Prostate* is not a treatment-specific tool; therefore, by assessing discrimination within treatment sub-groups, its discriminatory performance will inevitably be reduced. Nonetheless, PREDICT *Prostate* performed significantly better in predicting ACM and PCSM in most treatment groups. We also confirmed that adding in biopsy data to the model improved the performance though this effect was marginal in addition to the other variables already included. Using PPC as a continuous variable maximises the use of prognostic information, and this parameterisation did lead to marginally superior discrimination for ACM.

The primary utility of PREDICT *Prostate* will be in men for whom conservative management and radical treatment might both be appropriate options, for whom the decision is most difficult. Abundant literature demonstrates that decision aids contribute to more knowledgeable and informed patients and that they can improve clinician-patient communication [25, 26]. Therefore, the model may have wide potential applications in informing patient, clinician and multi-disciplinary team decision-making to reduce both over- and under-treatment. Formal clinical impact assessments are also crucial to show face and functional validity, and these are underway with PREDICT *Prostate* [27]. Future research endeavours could assess what impact the use of the model might have on actual treatment practices and compare this model with prognostic biomarkers, or radiological prognosticators. Over time, additional parameters can be incorporated into this base model, or the model itself be updated, should new variables be shown to have independent prognostic effects [28].

More recent efforts in prognostic tools have sought to utilise novel genomic or biological markers to generate prognostic estimates. However, most established genomic tools such as Prolaris CCP and Oncotype DX GPS have predominantly been tested against shorter-term outcomes or in treatment-specific cohorts [29, 30]. Where they have been assessed against PCSM, concordance has been very similar to our model—for example, the Decipher genomic classifier alongside CAPRA showed an AUC of 0.78 (95% CI 0.68–0.87) for 10-year PCSM following prostatectomy [31]. Direct comparison with PREDICT *Prostate* is not possible without a head-to-head or combined study, but the value of such expensive tests do need to be re-assessed in the context of optimised clinical multivariable models [32]. In this context, we would welcome collaborations or independent studies on the value of adding genomic classifiers to future iterations of PREDICT *Prostate*.

This study has numerous strengths, given the large sample size, long follow-up and high completeness of data in PCBaSe [33]. However, we recognise limitations inherent to using registry data. Seventeen per cent of men were excluded due to missing data, and we cannot exclude this, introducing some bias. A large proportion of men within this validation dataset had low-grade disease, such that PCa mortality rates were relatively low which may affect discriminatory performance. Men diagnosed within the inclusion period may also not be representative of contemporary practice with changes in PCa diagnosis and treatment. For instance, we recognise that primary hormone therapy is now rarely used in the context of non-metastatic PCa; hence, we included sub-group analyses within other treatment groups. We also appreciate that multi-modal therapies are increasingly used in higher risk cases, which we were not able to assess in this study due to the inclusion dates and data availability limitations of our datasets. Another particular concern is the lack of information from magnetic resonance imaging (MRI). However, the current focus for MRI is on tumour detection rather than prognostication and it is unknown if MRI lesion characteristics (Likert or PIRAD scoring) have any bearing on survival. Our model also cannot account for subsequent transitions to different treatments. However, in our UK dataset, conversions to active treatment were less than 6% across total follow-up [3]. We also recognise the lack of T-stage sub-classification, which is a key parameter in 2 of the existing models we made comparisons to. However, it is accepted that T stage is often inaccurately assigned in localised disease [34]. We also recognise that other endpoints of interest exist, particularly the development of metastases and commencement of hormone therapy. The model is untested against these endpoints, but calibrated against the more robust endpoint of death.

A key issue going forward is the validation of this model in non-Caucasian and screened populations. Although the original paper re-tested the model in Singaporean men, PREDICT *Prostate* remains untested in men of African descent or other ethnicities. Independent validations within screened populations, and within other prospectively collected or randomised datasets, would also be helpful and should be encouraged. Finally, we recognise that other nomograms are available, against which direct comparisons would be very insightful. These were not possible within the design of this study, or the limitations of this data, particularly with regard to comorbidity.

## Conclusions

This large external validation demonstrates the robustness of PREDICT *Prostate.* PREDICT *Prostate*, available as a free-to-use web tool [4], has the potential to significantly improve shared decision-making for PCa management, particularly the choice between conservative management and radical treatment. Further, independent external validations are encouraged, especially in populations of different ethnicities.

## Supplementary information

**Additional file 1.** Supplementary files.

**Additional file 2.** Data request and study outline form to PCBaSe.

## Data Availability

The datasets used in the current study can be made available at a remote server with export of aggregated data only. The application will be considered by the Prostate Cancer data Base Sweden reference group and The Research Ethics Board in Uppsala. Please contact author PS.

## Abbreviations

ACM: All-cause mortality
AJCC: American Joint Committee on Cancer
AUC: Area under the curve
CAPRA: University of California San Francisco cancer of the prostate risk assessment score
c-index: Concordance index
EAU: European Association of Urology
NCCN: National Comprehensive Cancer Network
NICE: National Institute for Health and Care Excellence (UK)
NPCM: Non-prostate cancer mortality
O:E ratio: Observed to expected ratio
PCa: Prostate cancer
PCBaSe: Prostate Cancer data Base Sweden
PCSM: Prostate cancer-specific mortality
PIRADS: Prostate imaging reporting and data system
PPC: Proportion of positive cores
PSA: Prostate-specific antigen
T stage: Tumour stage
UK: United Kingdom
95% CI: 95% confidence interval
