# Supplementary material for: Comparative performance and external validation of the multivariable PREDICT Prostate tool for non-metastatic prostate cancer: a study in 69,206 men from Prostate Cancer data Base Sweden (PCBaSe)
Source: BMC Med. 2020 Jun 16;18:139. doi: 10.1186/s12916-020-01606-w (PMC7296776; doi:10.1186/s12916-020-01606-w)
Supplement: Supplementary file 1 — Additional file 1. Supplementary files. [file 12916_2020_1606_MOESM1_ESM.docx]

**Additional File 1**

**Title**Comparative performance and external validation of the multivariable PREDICT *Prostate* tool for non-metastatic prostate cancer: A study in 69,206 men from Prostate Cancer data Base Sweden (PCBaSe)

**Authors**

David Thurtle, Ola Bratt, Pär Stattin, Paul Pharoah* & Vincent Gnanapragasam*

|  | **UK** |  |  |  | **Sweden** |  |  |  |
| --- | --- | --- | --- | --- | --- | --- | --- | --- |
| EAU Risk Group | Conservative (AS/WW) | RT | RP | ADT | Conservative  (AS/WW) | RT | RP | ADT |
| Low | 433 | 220 | 80 | 64 | 8501 | 1825 | 7066 | 375 |
| Intermediate | 776 | 1045 | 526 | 579 | 8060 | 5015 | 11209 | 3004 |
| High | 176 | 1192 | 389 | 1583 | 3823 | 5066 | 2661 | 12601 |
| Total | 1385 | 2457 | 995 | 2226 | 20384 | 11906 | 20936 | 15980 |

Table S1 – Breakdown of patients within the UK model development cohort and Swedish PCBaSe cohort, according to EAU risk stratification group, and primary treatment type. (AS = active surveillance, WW = watchful waiting, RT = radiotherapy, RP = radical prostatectomy, ADT = androgen deprivation therapy)


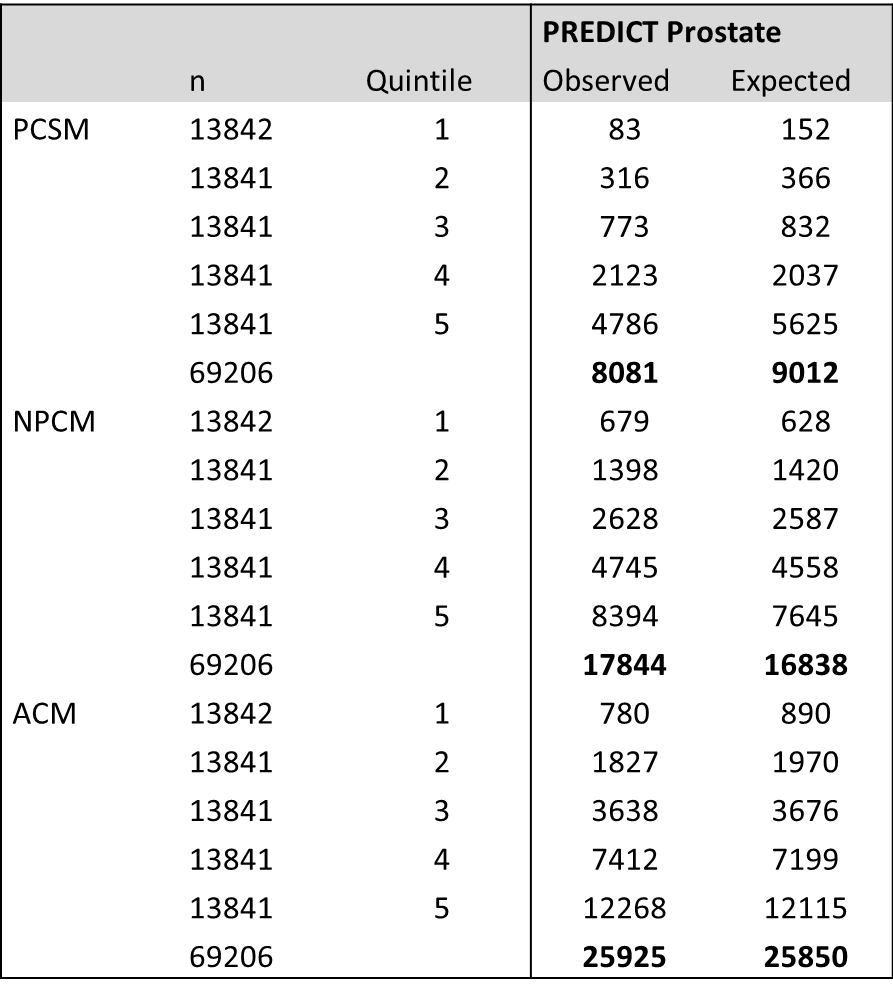


Table S2 – Overall calibration of the PREDICT Prostate model. Observed numbers of deaths are compared to expected numbers of deaths predicted by the model. (PCSM = Prostate cancer specific mortality. NPCM = Non prostate cancer mortality. ACM = All cause mortality)


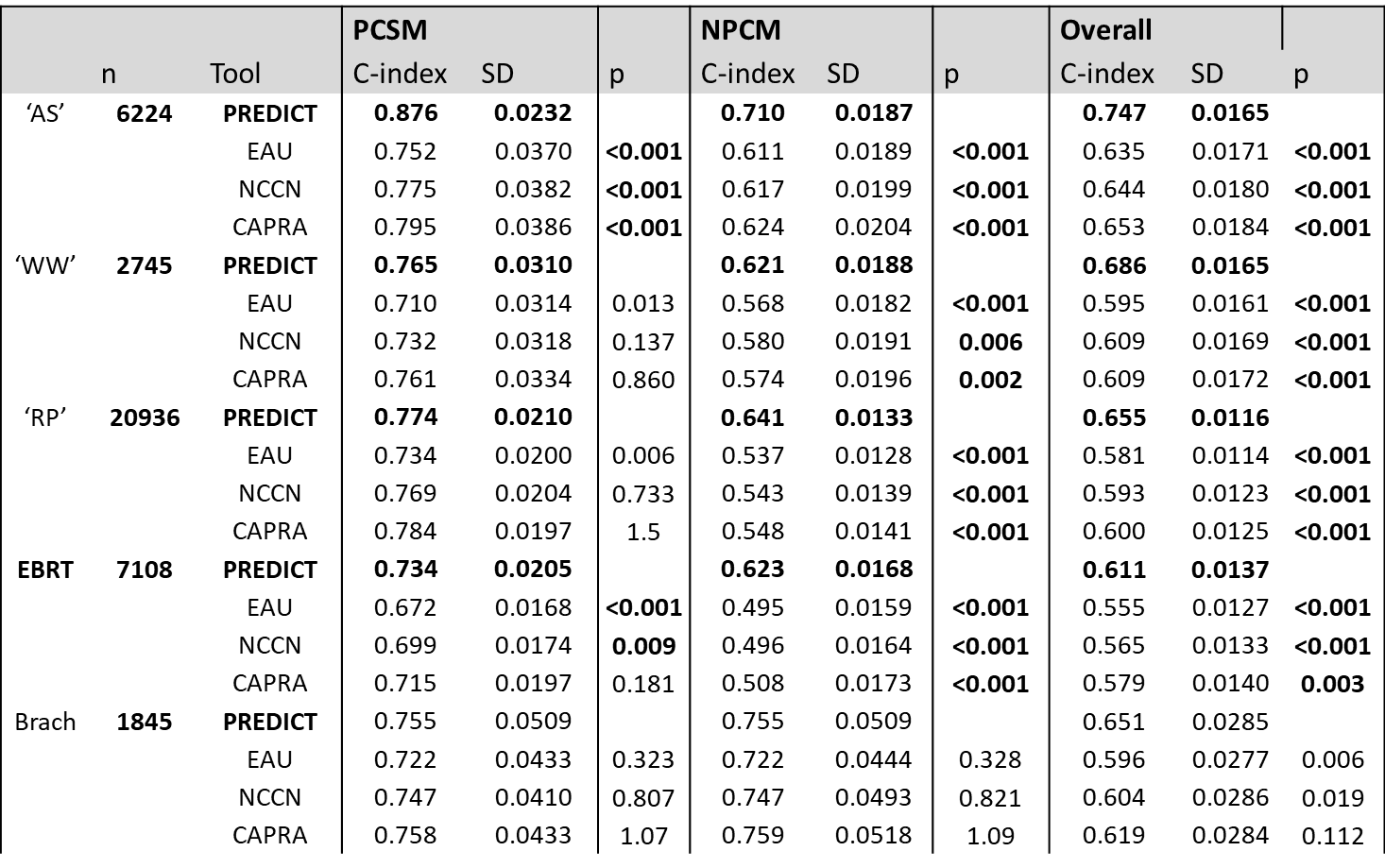

Table S3 – Discrimination within treatment sub-groups and comparison to existing models


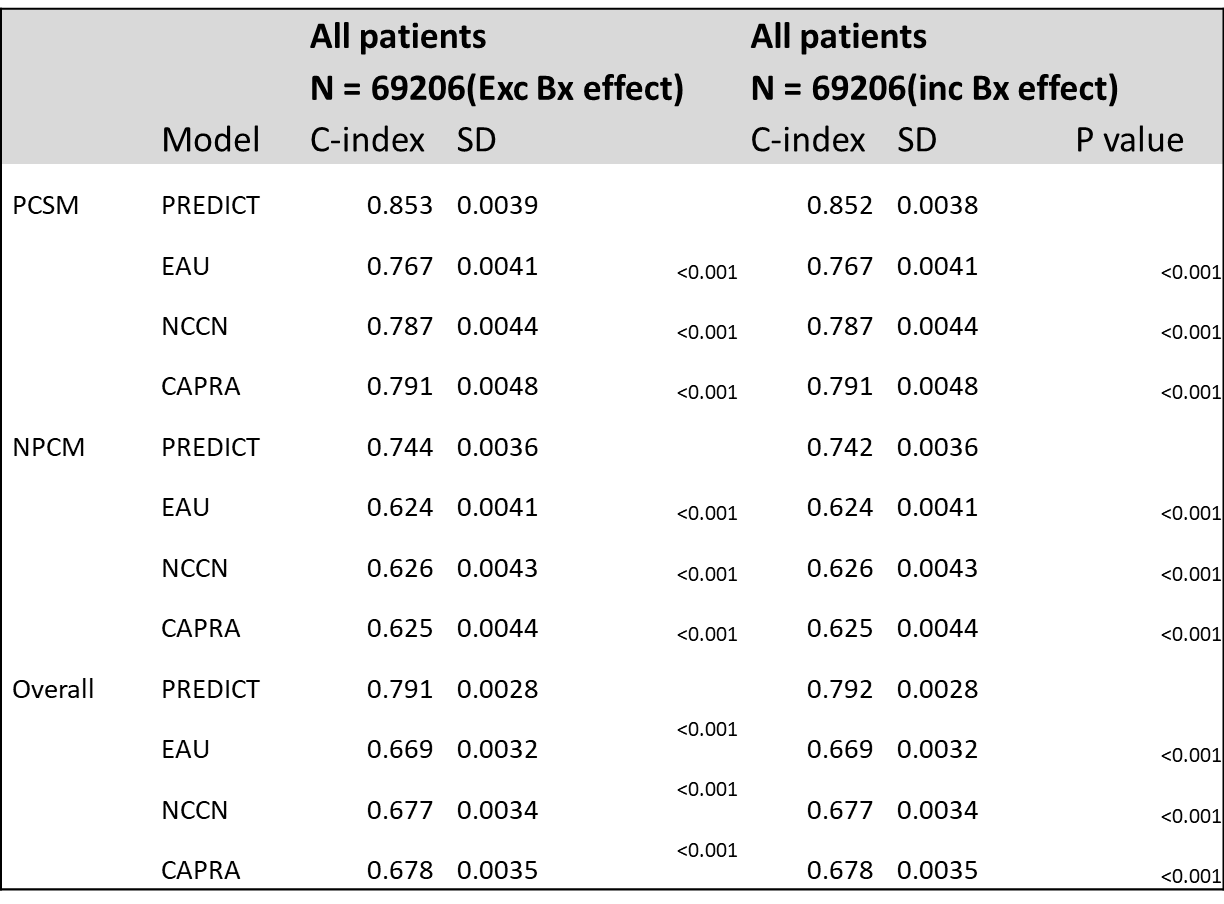

Table S4 – Comparison of discrimination within the PCBase cohort, using the PREDICT prostate model excluding and including the biopsy parameter.

|  | **Exc Bx Info**  **C-index** | **Biopsy50**  **C-index** | **PPC FP C-index** |
| --- | --- | --- | --- |
| **PCSM** | 0.8608 | 0.8629 | 0.8559 |
| **NPCM** | 0.7374 | 0.7337 | 0.7322 |
| **ACM** | 0.7786 | 0.7830 | 0.7840 |

Table S5 – Discrimination across the biopsy sub-cohort using three different biopsy categorisation methods: Excluding biopsy information completely (Exc Bx Info), Using a cut-off of >=50%PPC (Biopsy50), or using a fractional polynomial of PPC (PPC FP).
PPC – Percentage of positive cores


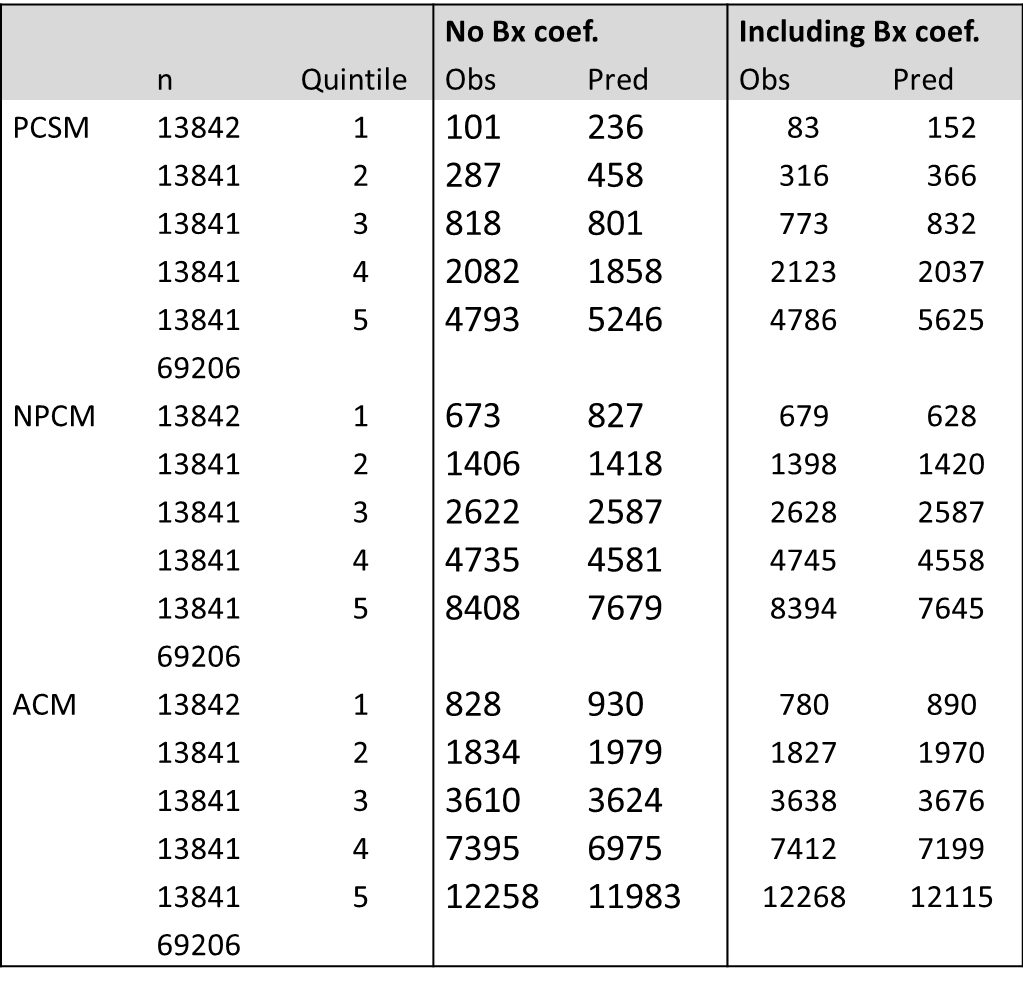


Table S6 – Assessment of calibration within the PCBase cohort across quintiles of risk. Comparison is made between the PREDICT prostate model excluding and including the biopsy parameter.

Figure S1 – Calibration curves demonstrating observed and expected 15-year probability of death across quintiles or risk for PCa death (left), non-PCa death (centre) and any cause death (right) within the biopsy sub-cohort, using no biopsy parameter (top row), the 50% cutoff parameter (middle row) and the PPC FP parameter (bottom row).
