# Supplementary material for: Comparative performance and external validation of the multivariable PREDICT Prostate tool for non-metastatic prostate cancer: a study in 69,206 men from Prostate Cancer data Base Sweden (PCBaSe)
Source: BMC Med. 2020 Jun 16;18:139. doi: 10.1186/s12916-020-01606-w (PMC7296776; doi:10.1186/s12916-020-01606-w)
Supplement: Supplementary file 2 — Additional file 2. Data request and study outline form to PCBaSe. [file 12916_2020_1606_MOESM2_ESM.docx]

**Additional File 2 – Data Request and Study Outline Form to PCBaSe**

**External validation study of ‘PREDICT: *Prostate’:* an individualised pre-treatment prognostic model for non-metastatic prostate cancer**

**April 2018**

**Project leads:**
Mr Vincent Gnanapragasam, Senior Lecturer and Honorary Consultant Urologist, Department of Surgery, University of Cambridge, UK

Mr David Thurtle, Clinical Research Associate and Urology Trainee, Department of Surgery, University of Cambridge, UK

Prof Paul Pharaoh, Professor of Cancer Epidemiology, Department of Public Health and Department of Oncology, University of Cambridge, UK

**Background**Prognostic stratification is the cornerstone of management for non-metastatic prostate cancer (PCa). However, no high-quality individualised model for survival exists. Available prognostic models are predominantly built around single-centre outcome data and short term surrogates for survival amongst populations that are heavily radically treated and PSA-screened.

Using a prospectively maintained database of 10,089 men from the UK National Cancer Registration and Analysis Service, we have developed an individualised prognostic model for cancer-specific and overall survival called PREDICT: *Prostate*. This is novel in that it uses and predicts long term (10 and 15 year) survival outcomes, provides individual percentage survival estimates rather than group estimates or relative risks, and represents real-world data from a non-screened, primary diagnostic cohort. Importantly, it also incorporates estimated treatment effects as it is designed for use before treatment. The methodology is also novel compared to other PCa models in using fractional polynomials and modelling both cancer-specific and non-cancer outcomes within a competing risks framework.

PREDICT: *Prostate* combines age, PSA, histological grade group, biopsy involvement, stage, primary treatment type and comorbidity to predict 10 and 15-year outcomes. Within a split cohort UK validation the model demonstrated good discrimination with AUC 0.83 (95%CI: 0.80-0.85) and 0.83 (95%CI: 0.81-0.84) for 10-year PCSM and Overall mortality respectively. This significantly outperformed existing models (p<0.001) and calibration was good with no significant difference between predicted and observed PCa-specific (p=0.19) or overall deaths (p=0.43). External validation was also attempted within a small Singaporean cohort, where results were also promising with <1% differences in actual and predicted deaths and AUC of 0.84 (95%CI 0.80-0.87) and 0.78 (95%CI 0.75-0.80) for PCSM and overall mortality respectively. However, these data had much shorter median follow-up of 5.1 years, and were missing information on comorbidity.

**Objectives:**- To evaluate the performance of PREDICT: *Prostate* within the PCBase cohort
- Compare PREDICT: Prostate to existing models within the PCBase cohort

**Justification and Methods**External validation in independent cohorts, ideally in a different location, is vital to demonstrate generalizability and accuracy of a multivariable prognostic model. It is also a requirement for model endorsement [6]. Performance within the original data, even a randomly split dataset, may well be optimistic [32]. The PCBase cohort represents the best available population data for PCa and importantly it contains all the variables used within our model. PCBase is also maintained, and updated with vital status information in a manner that is similar to our original cohort. External assessment within this dataset will be informative and represent the best possible methodology for model validation[33]. Using the statistical methods outlined beneath, 10 and 15-year mortality estimates will be compared to observed outcomes within the cohort. Comparison of model performance between cohorts will be assessed and recalibration or updating of the model performed if necessary. The study will be published in the medical literature and the results will be used to inform a free an online tool which is currently under development.

Adequate validation requires that we use the fully specified existing prognostic model (both the selected variables and their coefficients) to predict outcomes for the patients in the second dataset[33]. Data analyses will all be performed in Stata™ 14. The cohort will be tidied to match the variables and data setup used in PREDICT: *Prostate*. Only men with intact data on age, PSA, gradegroup, t-stage and primary treatment type will be included as these are integral variables for the model. Completeness of comorbidity information will be reviewed and included if possible. A summary of missing data and exclusions will be generated.

Beta coefficients for each prognostic factor in the model will be applied to derive prognostic indexes for PCa specific mortality (PCSM) and non-PCa mortality (NPCM) for each patient. These will be used in combination with the model’s baseline hazard functions and time-at-risk to create individual estimates of unadjusted PCSM and NPCM at 10 and 15 years (or maximum available follow-up, if shorter). These estimates will be converted into survivals and used to calculate overall mortality by adjusting for the competing risks between the two causes of death. Finally, adjusted 10 and 15 year predictions of cumulative PCSM and NPCM will be generated using the proportions of cause-specific mortality multiplied by overall mortality.

Across the cohort, comparison will be made between the estimated and observed numbers for cause-specific death. Calibration will be assessed by Chi-squared goodness of fit across quintiles of risk using the method of May and Hosmer [15]. Calibration will also be assessed across sub-groups including those only treated by active surveillance, conservative management or radical treatment. The ability of the model to discriminate PCSM, NPCM and overall mortality will be assessed by calculating the area under the ROC curve. Comparison of discrimination using PREDICT: *Prostate* will be compared to existing risk stratifications derived from the available data – namely the EAU, NCCN and UCSF CAPRA scores – using the method of DeLong[34].

**Timelines**Month 1: Data receipt and tidying
Month 2: Missing data analysis and report.
Month 3-4: Model application and accuracy assessments
Months 5-6: Review and model recalibration if necessary

**Deliverable outcomes**-High impact factor publication exploring the external validation of PREDICT: Prostate
-Informing and improving a prognostic model which has significant potential for clinical impact and widespread uptake.

**Summary**PREDICT: *Prostate* has the potential to significantly improve PCa management through informing patients and clinicians about prognosis. External validation in a geographically independent cohort will significantly improve confidence in the model, and should improve reliability and stability. Through this collaboration we would anticipate high impact publications and further refinements to our tool which will be available publicly online.
